# Antiretroviral Treatment Start-Time during Primary SIV_mac_ Infection in Macaques Exerts a Different Impact on Early Viral Replication and Dissemination

**DOI:** 10.1371/journal.pone.0010570

**Published:** 2010-05-11

**Authors:** Pierre Sellier, Abdelkrim Mannioui, Olivier Bourry, Nathalie Dereuddre-Bosquet, Benoit Delache, Patricia Brochard, Julien Calvo, Sophie Prévot, Pierre Roques

**Affiliations:** 1 Division of ImmunoVirology (SIV), Institute of Emerging Diseases and Innovative Therapies (IMETI), CEA, Fontenay-aux-Roses, France; 2 UMR E1, University Paris Sud XI, Orsay, France; 3 Hôpital Lariboisière, Assistance Publique-Hôpitaux de Paris, Paris, France; 4 Service d'Anatomie et Cytologie Pathologiques, Hôpital Antoine Béclère, Assistance Publique-Hôpitaux de Paris, Clamart, France; University of Pittsburgh, United States of America

## Abstract

**Background:**

The time of infection is rarely known in human cases; thus, the effects of delaying the initiation of antiretroviral therapy (ART) on the peripheral viral load and the establishment of viral reservoirs are poorly understood.

**Methodology/Principal Findings:**

Six groups of macaques, infected intravenously with SIV_mac251_, were given placebo or antiretroviral therapy to explore reservoir establishment; macaques were treated for 2 weeks, with treatment starting 4 hours, 7 or 14 days after infection. Viral replication and dissemination were measured in the gut (rectum), in the lung and in blood and lymphoid tissues (peripheral lymph nodes), by quantifying viral RNA, DNA and 2LTR circles. We used immunohistochemistry (CD4 and CD68) to assess the impact of these treatments on the relative amount of virus target cells in tissue. Treatment that was started 4 hours post-infection (pi) decreased viral replication and dissemination in blood and tissue samples, which were assessed on day 14 (RNA/DNA/2LTR circles). The virus remained detectable and lymphoid tissues were activated in LN and the gut in both placebo- and ART-treated animals. Viral RNA in plasma continued to be lower in macaques treated seven days after infection; however, this was not the case for viral DNA in peripheral blood mononuclear cells. There was a small but significant difference in RNA and DNA levels in tissues between placebo- and ART-treated animals on day 21. When started 14 days after infection, treatment resulted in a limited decrease in the plasma viral load.

**Conclusions:**

Treatment that was started 4 hours after infection significantly reduced viral replication and dissemination. When started 7 days after infection, it was of slight virological benefit in peripheral blood and in tissues, and treatment was even less effective if started 14 days pi. These data favor starting ART no longer than one week after intravenous SIV_mac251_ exposure.

## Introduction

Antiretroviral therapy (ART) inhibits viral replication, but does not eradicate cellular reservoirs of the virus. Recommendations from the U.S. Department of Health and Human Services on post-exposure prophylaxis (PEP) favor the use of ART, through two nucleosidic reverse transcriptase inhibitors (NRTIs) and a protease inhibitor (PI) or efavirenz for 2-4 weeks, within three days of exposure to HIV [1]. French guidelines recommend starting prophylaxis treatment (using two NRTIs plus a PI) within four hours of exposure (Yeni P. *et al.*, www.sante.gouv.fr [2]). These recommendations are based on experiments in macaques challenged with simian immunodeficiency virus (SIV), mimicking the acute human infection; they suggest a greater benefit for PEP if initiated within 36 hours, compared with 72 hours after exposure [3], [4]. Nevertheless, being given HIV PEP within the optimal prescription window is a rare event, as most potentially exposed patients present 12 hours or even 24 hours after viral exposure [5]. It is unclear at what point PEP is no longer beneficial, and there is an absence of data on whether there is a clear benefit to PEP being initiated more than 48 hours after exposure. The precise virological and immunological consequences of these delays are poorly understood and it remains unclear whether delayed PEP actually leads to better progression of the infection. Therefore, defining the ideal periods for HIV PEP treatment, through the study of deep tissues in the macaque model, is a major concern.

Our main objective was to investigate the impact of ART treatment start time on viral spread during primary infections. The combination of AZT-3TC (zidovudine, lamivudine, 4.5 mg/kg, 2.5 mg/kg respectively; administered subcutaneously), and IDV (indinavir 60 mg/kg administered *per os*), administered twice-a-day for 28 days and started as early as 4 h after IV inoculation with 50 AID_50_ of SIV_mac251_, does not prevent infection; however, virus loads in plasma remained undetectable in most of the animals during the treatment period [6]. Here, we investigated to which extent the ART start time affects viral replication and dissemination during the primary infection.

We therefore evaluated the efficacy of the AZT/3TC/IDV combination, initiated 4 hours post-infection (pi) or delayed to before or after the viraemia peak (7 days pi, 14 days pi), on immunological and virological variables, both in peripheral and deep compartments. Treatment initiation times were chosen according to French PEP guidelines (4 hours), periods before acute CD4+ cell loss [7] and associated with complete protection in another macaque model [8] (7 days pi), or periods following CD4+ T cell loss (14 days pi). Previous macaque models infected intravenously with SIV [4] showed that courses of ART that are shorter than 28 days confer incomplete protection. We therefore investigated the effects of administering 2 NRTI plus an unboosted and weak PI over a 14-day period; the PI selected was a frequently recommended protease inhibitor at the time of the study in 2004. This thus allowed an extensive study of viral replication and dissemination in tissues.

We monitored plasma viral RNA, total viral DNA and 2LTR circle levels in peripheral blood mononuclear cells (PBMCs), lymph nodes, rectum and lung, to study viral replication and dissemination in detail. We studied the effects of infection on viral target cells (CD4+ T cells or CD68+ macrophages) in tissue through immuno-histochemistry (IHC), to precisely define the most effective and the ultimate treatment start time able to reduce viral dissemination in this model.

## Results

Twenty-eight cynomolgus macaques were infected with 50 AID50 of SIV_mac251_, and were then treated with a placebo, or the same combination of antiviral drugs described above (AZT, 3TC and IDV), initiated at 4 hours pi (10 animals, 5 ART-treated, 5 placebo-treated, group H4-D14), on day 7 pi (10 animals, 5 ART-treated, 5 placebo-treated, group D7-D21) or on day 14 pi (8 animals, 5 ART-treated, 3 placebo-treated, group D14-D28). Animals were killed 14 days after starting treatment.

### Delayed treatment during primary infection remained beneficial, due to a reduction in the plasma viral load and the prevention of a significant decrease in circulating CD4+ T cells

**Figure 1:**
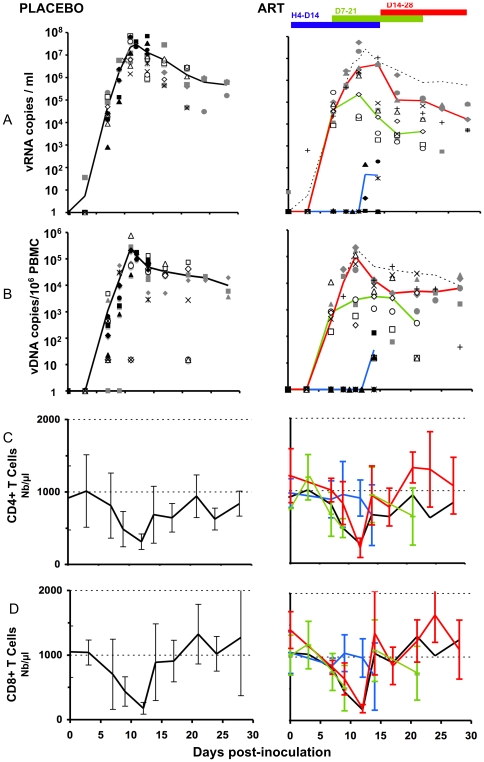
Peripheral viral loads and changes in CD4, and CD8 T cells in placebo- and ART-treated animals. Placebo animals are shown on the left side (median in dark line), and treated animals, on the right side of the figure. Values for animals are given as a filled symbol and a star (group H4-D14); open symbol and x (group D7-21); grey symbols and + (group D14-28). Median curves are shown according to the date of treatment initiation, with the black or dotted line for placebo and blue, green, and red lines for the H4-D14, D7-21 and D14-28 groups, respectively. A) Plasma viral load; vRNA copies/ml of plasma. B) Total viral DNA in PBMCs; vDNA copies/10^6^ PBMC. C) CD4 changes in absolute number (mean±SD) D) CD8 changes in absolute number (mean±SD).

As expected, infection in placebo-treated groups induced a peak in the plasma viral load and in viral DNA in the PBMCs between day 13 and 14 (**Figure 1A, 1B**). CD4 levels in peripheral blood in these placebo-treated animals were significantly depleted, with the nadir occurring on day 13 (Median 30% of the baseline, p = 0.0117). This was followed by a rebound to 60–70% of original levels (**Figure 1C**) and, in some animals, CD4 levels returned to base line values on day 28 (not shown). Note that during the initial acute phase, viral infection induced a significant decrease in CD8+ T cell counts (down to 5% of baseline values, p = 0.0117); however, in contrast to CD4 T cells, this was followed by a large early rebound in CD8 T cell counts on day 15 or 17. Thus, CD8 T cell counts increased to 150% of baseline values between day 21 and 28 (**Figure 1D**). This rebound is commonly associated with immune system activation and the detection of anti-HIV specific CD8+ cytotoxic T cells [6], [9].

The plasma viral load (**Figure 1A** right, blue line median) and total SIV DNA in PBMCs were significantly lower (p<0.01) in animals in which treatment was started 4 hours after infection than in placebo-treated animals, as previously reported [6], [10] (**Figure 1B** right, blue line median). Despite significant variability, circulating CD4+ (908±20 cells/mm^3^ at day 0 versus 708±537 cells/mm^3^ at day 14) and CD8+ T lymphocyte counts remained relatively stable (mean = 772±374 cells/mm^3^ at day 0 versus 857±828 cells/mm^3^ at day 14; **Figure 1C, 1D**). Circulating CD4+ and CD8+ T lymphocyte counts were significantly higher in PEP-treated animals (4 hr, day 7 and day 14 pi) than in placebo-treated animals (p = 0.007 and 0.02, respectively).

If delayed to 7 days pi, before the viral peak, the treatment, compared with placebo, exerted a significant impact on SIV RNA in plasma (p = 0.016), but not on SIV DNA in PBMCs (**Figure 1A, 1B** **,** green line). The circulating CD4+ T lymphocyte level on day 21 pi was not significantly different from that on day 7 (798±393 cells/mm^3^ and 750±300 cells/mm^3^ respectively). The CD8+ T lymphocyte count in treated animals (green) was not significantly greater (1000±652 cells/mm^3^) on day 21 pi (**Figure 1D**). The differences in circulating CD4+ (or CD8+) T lymphocyte counts between animals treated from day 7 to day 21 (green line) and placebo-treated animals (black line) did not reach significance.

Treatment of the last group (D14-D28) was started after plasma viral loads peaked on day 14 pi. Treatment significantly affected plasma viral load on day 28 (p = 0.025), but not SIV DNA in PBMCs, if compared with placebo (**Figure 1A, 1B**, red line). Before treatment, T cell counts of these animals were almost identical to those in control groups. From day 14, CD4+ cell counts increased to slightly above the baseline in four of five animals (**Figure 1C**, red line). CD8+ T lymphocyte counts did not significantly differ between day 14 and day 28 (mean 1155±658, and 1292±465 cells/mm^3^ respectively; p = 0.63; **Figure 1D**, red line). The differences in circulating CD4+ (or CD8+) T lymphocyte counts between animals treated 14 days pi and placebo-treated animals did not reach significance, but a strong trend was observed in relation to the CD4+ T cell count (p = 0.095).

### Impact of antiviral therapy start time on viral replication and dissemination in deep tissues

**Figure 2:**
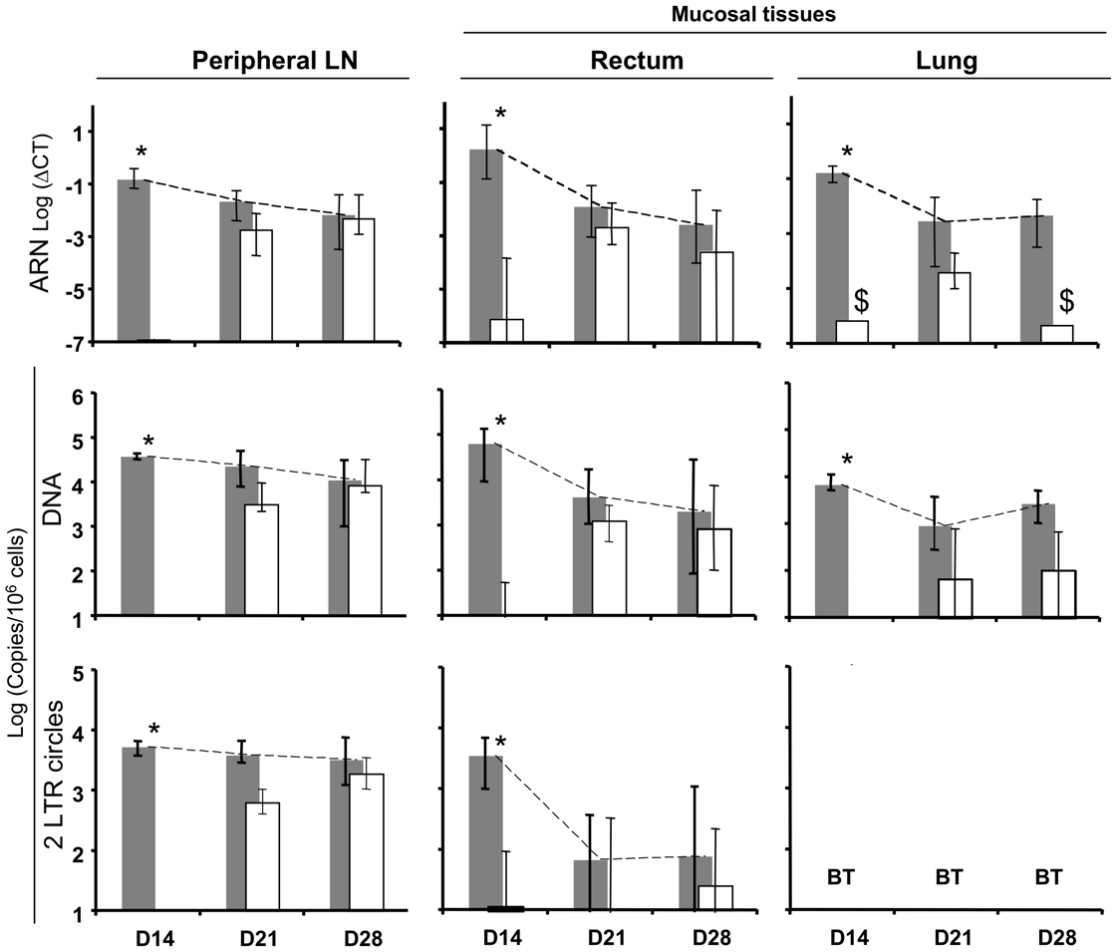
Change in viral loads in tissues in animals given placebo or treatment over the course of 14 days. Viral loads were expressed using RNA Log (ΔCT), DNA and 2LTR Log(Copies/10^6^ cells) at the top, middle and at the bottom of the figure, in peripheral LN, rectum, and lung (left side, middle, and right side), respectively. LN: lymph nodes; placebo animals (Gray bars), treated animals (white bars). Vertical bars represented the 95% confidence interval (95% CI). 2LTR values in the lung were below the threshold and were listed as BT. $ viral RNA was detected in only 1 animal in ART-treated animals on day 14 and day 28. * Significant differences between placebo and treated animals.

We extended our analysis to peripheral lymph nodes (LN) and mucosal tissues, to determine the spread of the virus more precisely, based on whether antiviral therapy was administered early or late. We used a combination of three viral markers – viral DNA (indicating dissemination), viral RNA (an indicator of viral replication and production), and 2LTR circles (indicating new infection) – to study, in detail, viral dissemination and the dynamics of viral replication in tissues [11]. We showed that early treatment (4 hours pi) significantly decreased both viral replication and dissemination for up to 5 level of magnitude compare to placebo in peripheral LN and mucosal tissues (rectum and lung, **Figure 2**). Treatments that were started seven days after infection continued to impact, although to a lesser extent, both viral replication and dissemination in peripheral LN and mucosal tissues (with geometric mean decreases in viral load for all combined tissues: for SIV RNA, 0.8× log_10_, p<0.05, for SIV DNA, 0.7× log_10_, p<0.05, respectively) (**Figure 2**). Viral dissemination was lower in mucosal tissues than in LN (p<0.02). Finally, in the LN, ART that was started on day 14 pi had no effect on viral replication/dissemination, with SIV RNA/DNA/2-LTR circles in LN remaining roughly unchanged. In the rectum and the lung, ART resulted in a slight, but not significant, decrease in SIV DNA (p = 0.06), whereas RNA was only detected in one macaque on day 28 (**Figure 2**). As in our previous study (Mannioui *et al.* [11]), there was a good correlation between the three markers in the various tissues, similar to that seen in PBMCs.

### Impact of antiviral therapy start time on target cells in deep tissues

**Figure 3:**
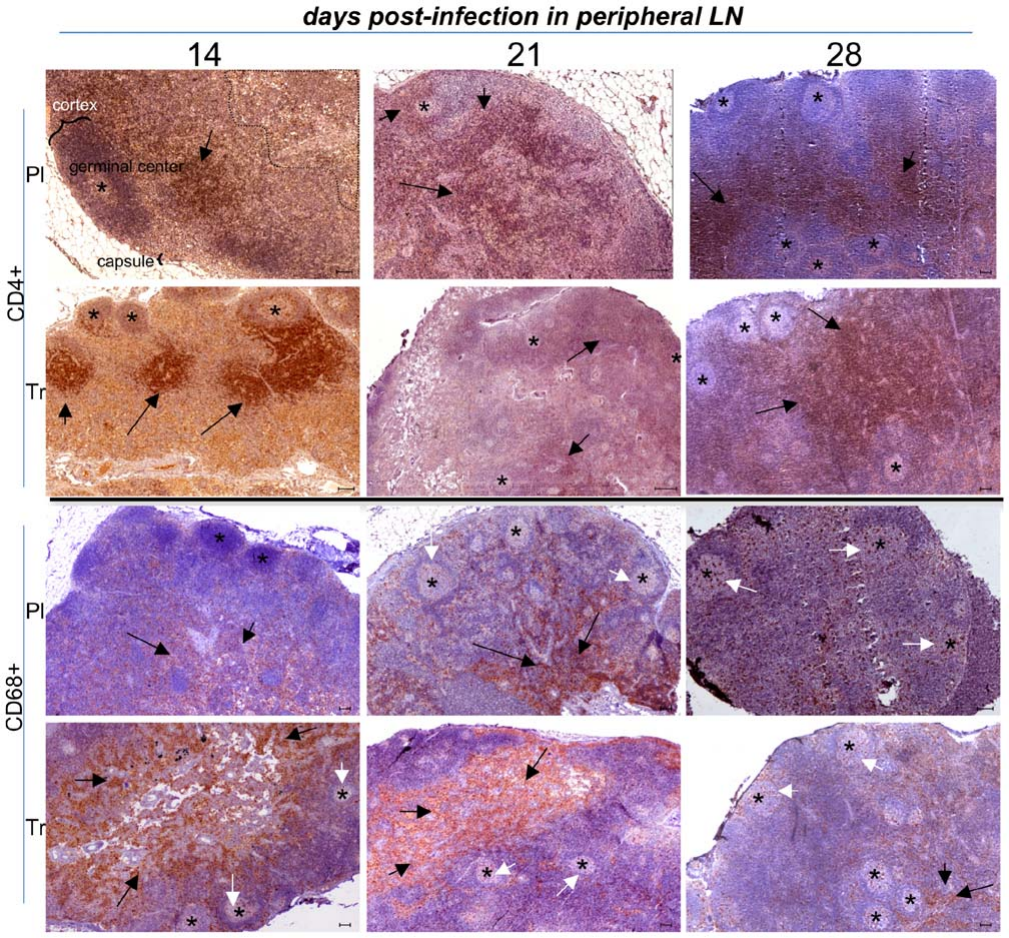
Low magnification of IHC stains of peripheral LN samples in placebo- and ART-treated animals. For clarity, we identified the germinal center (GC), cortex and capsule from a lymph node in one of the panels. The limit of the white pulp region is shown by a dotted line, and germinal centers are shown using stars. CD4+ staining surrounded lymphoid follicles (black arrows), whereas CD68+ staining was mainly localized in the white pulp (black arrows) and to a lesser extent in the GC (white arrows). Target cells were shown at the top (CD4+) and the bottom (CD68+) of the figure. For each pair of presented animals, placebo-treated animals were located above, and PEP-treated animals below. Animals killed on day 14, day 21, and day 28 were shown from the left to right of the figure. The horizontal bar on each panel corresponds to 100 µm. Each picture was representative of explored tissues and was cropped from a large image. Lymph node and GALT architecture organization is shown; specific staining is colored brown.

**Figure 4:**
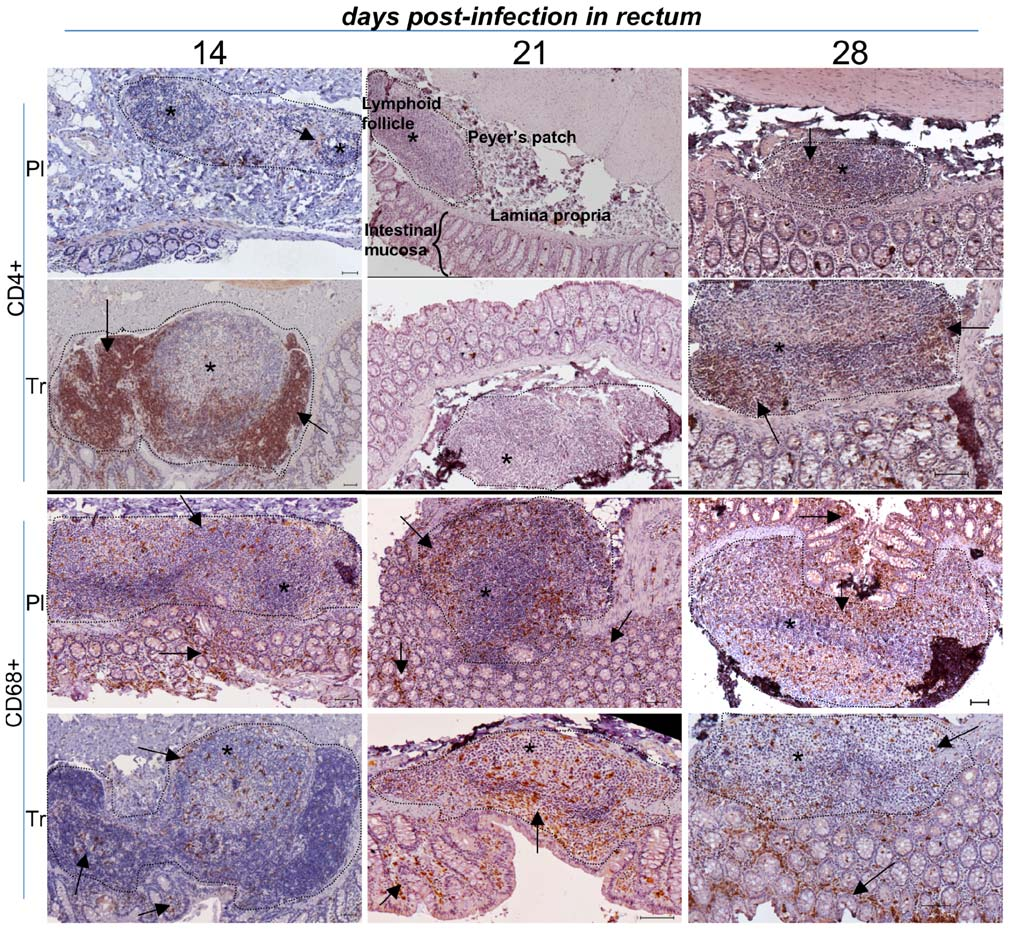
Low magnification of IHC stains of rectal samples in placebo- and ART-treated animals. The lymphoid-rich area (Payer's patch) was sectioned by a dotted line and stars indicated lymphoid follicles; rare CD4+ lymphocyte staining is highlighted by arrows. CD68+ staining is seen in both lymphoid follicles and interstitial zones (arrows). Presentation as in Figure 3.

**Figure 5:**
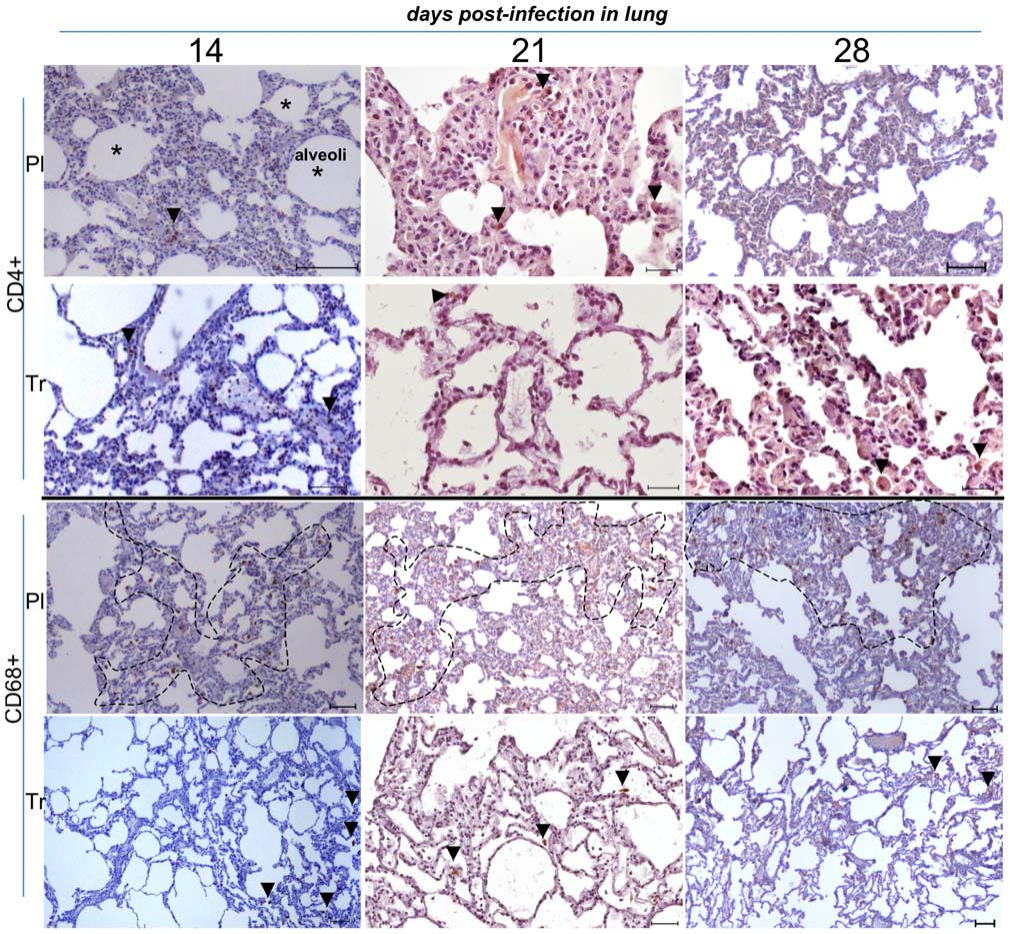
Low magnification of IHC stains of lung samples in placebo- and ART-treated animals. CD4+ staining was occasionally found in the lung (arrow). CD68+ (alveolar monocyte/macrophages) staining was greater in the placebo (dotted line) than in ART-treated animals (arrow). A random portion of the lung was taken from a large image. Presentation as in Figure 3.

The correlation between the level of viral replication in tissues and the “in situ” depletion of target cells is not well understood and is often not documented. Thus, we performed IHC in the peripheral lymph nodes (LN) (**Figure 3**), rectum (**Figure 4**) and lung (**Figure 5**).

**In Lymph nodes** (LN) from placebo-treated animals, IHC performed on day 14 pi showed hypertrophic B follicles surrounded by large T CD4+ areas localized in the cortex. Antiviral therapy that was started 4 hr pi did not result in CD4+ T cell depletion in lymph nodes (**Figure 3**). Despite a low viral load in tissues (**Figure 2**), we found hypertrophic B lymphoid follicles surrounded by enlarged CD3+ (not shown) and CD4+ T lymphocyte areas (arrows) in LN (**Figure 3**). In placebo-treated animals, a depletion of T CD4+ occurred on day 21, followed by a partial repopulation on day 28. We observed a progressive redistribution of CD68+ cells, from the medulla to the germinal center of follicles localized in cortex, in LN from placebo-treated animals; this redistribution occurred from day 14 to day 28. If started before peak viremia (day 7 pi), the treatment did not stop the depletion of CD4+ T lymphocytes on day 21 (**Figure 3**), and did not modify the distribution of CD68+ cells compared to placebo. Treatment that was started after peak viremia (day 14 pi) did not modify the level of depletion on day 21, but the slight preservation of CD4+ T cells in lymph nodes may be explained by a redistribution of these cells from peripheral blood (**Figure 3**). IHC showed an increase in CD3+ (not shown) and CD4+ T lymphocyte levels in peripheral LN, without reconstitution of the previous architectural structure (B follicles surrounded by hypertrophied CD4+ T lymphocyte areas). T zones in LN occurred in deep areas and were slightly disorganized. In the same PEP- treated animals, no differences were observed for the distribution of CD68+ cells.

**Figure 6:**
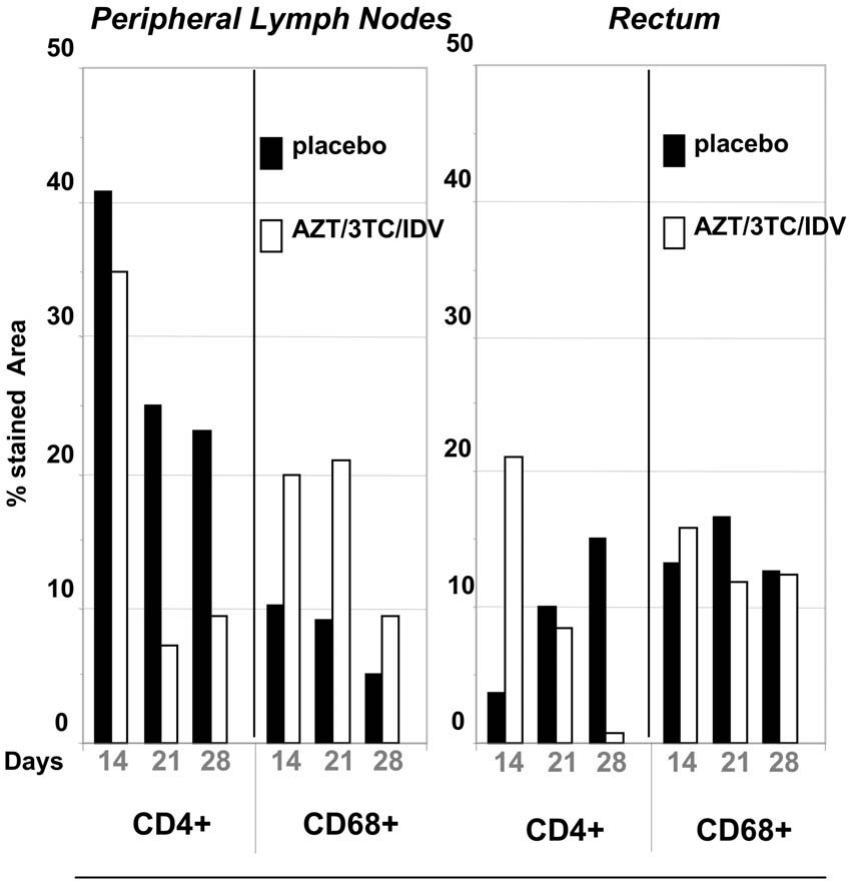
Percentage of the area stained for CD4+ and CD68+ cells (IHC) in peripheral LN and rectum, according to treatment groups and dates of initiation. Dark dot blots symbolized placebo-treated animals, and white dot blots, PEP-treated animals. The percentage of the stained area was determined by a quantification performed using two animals per group (see Material and methods).

Depletion **in rectal samples** in placebo-treated animals (day 14) seemed to occur earlier (**Figure 4**), with only few CD4+ T cells found in Peyer's patches. This early mucosal CD4+ T cell depletion has been previously described during acute HIV infection [12]. The initiation of antiviral therapy 4 hours after infection prevented CD4+ T cell depletion in rectal mucosae (**Figure 4**). However, despite a low viral load in tissues of PEP-treated animals (**Figure 2**) on day 14, we found hypertrophic B lymphoid follicles surrounded by enlarged CD3+ (not shown) and CD4+ T lymphocyte areas (arrows) in Peyer's patches in the rectum (**Figure 4**). CD4+ T cell depletion was clearly observed on day 21 in rectal mucosae from placebo-treated animals. Nevertheless, the delayed treatment, which was initiated before the viremia peak (day 7 pi), did not prevent CD4+ T lymphocyte depletion on day 21 (**Figure 4**). Surprisingly, CD4+ T cell depletion from placebo-treated animals was less pronounced in day 28 samples; this slight increase may be explained by redistribution from peripheral blood (**Figure 4**). CD68+ cells were found both in Peyer's patches and intestinal mucosae, regardless of the time of killing. Depletion of CD4+ T cells in day 14-day 28-treated animals on day 28 seemed greater than that in placebo-treated animals, whereas the number of CD68+ cells did not appear to be modified by treatment (see below Figure 6).

**In lung samples** from placebo-treated animals, the numbers of T CD4+ cells progressively decreased from day 14 to day 28 (**Figure 5**). CD68+ cells were identified on day 14, 21, and day 28, with no organized structure. The initiation of antiviral therapy 4 hours after infection prevented CD4+ T cell depletion in lung tissues (**Figure 5**). However, despite a low viral load on day 14 in tissues of treated animals (**Figure 2**), the number of CD4+ T lymphocytes was sparse (arrow heads); there were several cells expressing CD68+ (dotted area) (**Figure 5**). As expected, CD68+ mononuclear cells remained the predominant leukocyte population in the lung, if compared with CD3+ lymphocytes (staining not shown).

To obtain a more objective view of the changes according to time of the number of CD4+ and CD68+ cells in the LN and rectum, we quantified the percentage of the stained area using large fields (**Figure 6**). This showed a very distinct pattern of infection between peripheral LN and mucosal tissues. **In placebo animals** (black bars), the percentage of the area that was stained for CD4+ cells in peripheral LN was very high on day 14 but decreased on day 21 and day 28. A similar change according to time was observed for CD68+ cells. CD4+ levels in the rectum were very low on day 14, but there was a marked increase in the CD4+ stained area by day 28, whereas the area stained for CD68+ did not change over the course of our study. **Analysis of treated animals** (white bars) showed that the effects of treatment in peripheral LN and the rectum were complex and distinct, and changed according to tissues and timing. In peripheral LN, the area stained for CD4+ had not changed by day 14, but CD4+ depletion appeared to be greater on day 21 and 28 despite treatment. By contrast, the area stained for CD68 was greater in all PEP-treated animals than those treated for placebo at the same time. Treatment 4 hr after infection appeared to be effective against early CD4+ T cells depletion in the rectum; this efficiency was lost when treatment was started later (D7-D21, and particularly D14-D28). There was no difference in areas stained for CD68+ between placebo and PEP-treated animals, at whichever point macaques were studied.

## Discussion

We aimed to investigate the impact of either early, or delayed ART during primary intravenous infection of macaques with pathogenic SIV_mac251_. Interestingly, during this period, there is a clear relationship between peripheral and tissue viral loads both in placebo and PEP-treated animals for a given date. Our findings favor the initiation of ART before the viremia peak, even one week after infection. ART initiated after the peak of viral replication showed no impact on viral spread, due to early dissemination.

Very low levels of viral replication (SIV RNA) and dissemination (DNA) were found in mucosal tissues, if ART was started four hours after infection (below the threshold in four animals out of 5; Fig. 2). The immunological benefit of preventing CD4+ T cell depletion in LN and mucosal tissues is obvious, as for acute infections: the mucosa is the dominant site of infection, and the gastrointestinal tract/other mucosal tissues contain at least half of the body's T cells [13]. In macaques infected with SIV, intestinal CD4+ T cells are almost entirely depleted within three weeks of infection [14]–[16]. Despite a few studies on the most acute stages of HIV-1 infection in humans, it is likely that there is a similar large and rapid loss of intestinal CD4+ T cells during the early periods of infection [17]–[19].

ART started seven days after infection continued to have an impact on both viral replication (RNA) and dissemination (DNA) in the gastrointestinal tract. Moreover, viral dissemination was lower in mucosal tissues than in LN. Nevertheless, delayed treatment did not stop the depletion of CD4+ T lymphocytes in the rectum, as shown by results on day 21. Verhoeven D *et al*., treating SIV-infected rhesus macaques with PMPA+FTC with the same schedule (from 7 days pi), reported similar acute CD4+ T cell loss two weeks after infection in the jejunum [7].

ART that was started 14 days pi did not result in a significant decrease of SIV DNA and RNA in the rectum. Macaques treated with PMPA eight weeks after infection have slightly lower intestinal (samples from jejunum and colon) SIV RNA levels [20]. Despite a clear activation of the immune system and modification of lymph-node architecture, which is typical of this infection [21], CD4+T lymphocyte localization seemed to be more conserved and the number of CD4+ T lymphocytes remained higher in treated animals in LN and in gut associated lymphoid tissues (GALT) than in placebo-treated animals.

Observational studies in humans, along with limited and contradictory studies using animal models, suggest that there may be a window of opportunity for initiating ART to preserve mucosal CD4 T cells or to allow a complete repopulation. Mehandru *et al.* found that therapy failed to significantly repopulate mucosal CD4+ T cells in eight HIV-infected subjects who started ART early during primary infection [22]. Tincati *et al.* evaluated the kinetics of CD4+ T-cell decrease and ART-mediated immune reconstitution in the gastrointestinal tract of nine patients during the acute phase of HIV infection, by performing rectosigmoid colonic biopsies before and after six months of ART [23]. Time from symptoms to therapy ranged from 13 to 49 days (mean 26 days) and the regimen most often used was identical to the one used in this study (AZT+3TC+IDV). HIV DNA was only marginally reduced in the gastrointestinal tract; this was associated with persistent immunological impairment in GALT. By contrast, Guadalupe et al. demonstrated that one patient who started ART within six weeks of infection showed significant mucosal repopulation [24]. Verhoeven D *et al*., using rhesus macaques infected with SIV and treated with FTC (emtricitabine)/PMPA at 1 weeks of infection over a period of 30 weeks, showed, despite major suppression of viral RNA levels in GALT, that ART led to a restoration of CD4+T cell levels in this tissue, in comparison with placebo-treated animals [25], [7]. Thus, ART may have been initiated too late, as a severe (and partially not reversible) depletion of CD4+ T cells in GALT had already occurred; this contrasts with the rapid and complete restoration of CD4+ T-cell levels in GALT, which is observed when the same regimen is initiated one week pi [7]. This suggestion is supported by a report by George M.D. et al.; they studied mucosal CD4+T-cell restoration in a model of rhesus macaques intravenously infected with SIV_mac251_ and given PMPA or placebo six weeks after infection, with PMPA given over the course of 20 weeks [26]. They demonstrated a marked suppression of mucosal viral loads and rapid reconstitution of CD4+T cells, in GALT of animals receiving ART. Treatment initiated one week after infection, using our combination of drugs (AZT/3TC/IDV) and over a period too short to be fully effective, reduced viral dissemination, but had no effect on LN hyperplasia. Despite the impact on viral replication and dissemination in lymph nodes and mucosal tissues, we were unable to show a clear benefit toward target cell depletion in these tissues. It may be related to a lack of potency in the treatment, and/or to the short duration of the experiment. Previous analysis of B cell functionality and distribution in these animals on day 14 (early ART) showed that ART has only a minimal effect on early Ig production and B cell distribution; however, ART stops the increase in germinal center growth and other changes observed on day 28 (D14-28 ART) in mesenteric LN and spleen [27]. Taken together, these data support a limited but real immunological benefit associated with ART treatment, even if our understanding of the underlying mechanisms requires further study.

Limitations in our study included difficulty in accurately defining the significance of the number of target cells in animals, as we did not know the status (resting/activated) of these target cells, and difficulty in accurately determining the level of viral production, despite our PCR evaluation. As all the animals were killed, another limit of our study was the inability to study the natural long-term outcome of the animals, such as cellular repopulation by target cells in the various tissues (peripheral lymph nodes, rectum and lung).

The individual virological (reduction of total SIV DNA in lymphoid organs, despite lack of protection) and public health (decreasing the infectiousness of HIV in patients with acute HIV-1 infection) benefits of delayed therapy, initiated before the peak of viremia, even one week after infection, should be taken into account for persons seeking care 72 hours after HIV exposure. Further studies should include time points between 48 and 72 hours, a drug regimen resembling current HIV PEP, and long-term experiments with or without the cessation of treatment, to study the potential for long-term change in viral replication patterns and clinical outcome by stopping or limiting the primary infection [28].

## Materials and Methods

### Animals and Ethics Statement

Twenty-eight adult Mauritian cynomolgus macaques (*Macaca fascicularis*), weighing 4 to 6 kg, were housed in single cages within level 3 biosafety facilities. They tested negative for SIV, simian T-lymphotropic virus, herpes B virus, filovirus, simian retrovirus 1, simian retrovirus 2, measles, hepatitis B virus HBsAg, and hepatitis B virus HBcAb. Animals were housed and cared for in accordance with the European guidelines for animal care (“*Journal Officiel des Communautés Européennes*,” L358, 18 December 1986). All protocols used in this study were reviewed and approved by a regional animal care and use committee: “Comité Regional d'Ethique sur l'expérimentation animale Ile de France Sud”, with the goal of improving animal welfare and limit suffering. The animals were sedated with ketamine chlorhydrate (Rhone-Merieux, Lyon, France), before virus injection, blood sample collection, and before receiving treatment or placebo, as previously described [6].

### Virus inoculation

Macaques were inoculated, via the saphenous vein, using 50% of the animal intravenous infectious dose (50 AID50) of a cell-free virus stock of pathogenic SIV_mac251_ (provided by A. M Aubertin, Université Louis Pasteur, Strasbourg, France) in 1 ml of phosphate-buffered saline (PBS) [29]. We assessed the *in vitro* susceptibility of the virus stock to AZT, 3TC, and indinavir: each compound, given alone at a concentration of 100 nM, inhibited 56, 76 and 94%, respectively, of SIV_mac251_ replication in a human PBMC culture assay, whereas, in combination at 10 and 100 nM, inhibition reached 93% (10 nM) and was close to 100% (100 nM) in a similar assay [30].

### Treatment of animals

Twenty-eight animals were divided into groups of 3 to 5 macaques each, and were given placebo (Fig. 1A) or the combination of AZT (4.5 mg/kg of body weight), 3TC (2.5 mg/kg) subcutaneously, and indinavir (60 mg/kg) orally, using a nasogastric catheter, twice-a-day [6]. AZT and 3TC were mixed with 9% sodium saline, to obtain respective concentrations of 4.5 and 2.5 mg/ml; indinavir was mixed with 0.5% methylcellulose, to obtain a final concentration of 60 mg/ml. The animals received 1 ml/kg of each solution. Treatment was initiated at 4 hours, on day 7 or on day 14 after infection, and was maintained for 14 days. The animals being given placebo or ART were then killed on day 14, 21 or day 28.

### T-lymphocyte subset determination

PBMCs were analyzed by flow cytometry with a FACScan cytometer, using CellQuest software (Becton Dickinson), as described previously [6]. Briefly, we incubated 30 µl of whole blood for 30 min with anti-CD3 monoclonal antibody (MAb) (FN-18) (Biosource International, Camarillo, Calif.), anti-CD4 (L200R, Phycoerythrin (PE)), and anti-CD8 (B9.11; PE-Cyanine 5) monoclonal antibodies (BD Biosciences, San Jose, CA, USA). Fluorescein isothiocyanate (FITC) and PE-conjugated immunoglobulins G1 (Immunotech, Marseille, France) were used as controls.

### Plasma viral load determination

Viral RNA was quantified, as previously described [31]. Amplifications were performed in duplicate within an iCycler thermocycler (Biorad, Marnes-la-Coquette, France). The standard RNA template dilution, over 7 orders of magnitude, showed a correlation coefficient of up to 97%, with a sensitivity corresponding to at least 60 copies/ml.

### Tissue collection

Peripheral lymph nodes from three locations (axillary, inguinal, and iliac), lung, (as part of mucosa-associated lymphoid tissue), and rectum, (as part of the GALT), were collected at necropsy on days 14, 21 and 28 pi. Cell-associated virus loads were then quantified in these organs, in all animals in the study.

### Tissue RNA and DNA extraction

Tissue lysates were obtained after mechanical disruption of tissue samples in RA1 buffer (Macherey Nagel, Hoerdt, France) with a Precellys system, using 18 CK tubes with ceramic beads (Bertin technologies, Montigny-le-Bretonneux, France). The tissue lysate was then diluted to 30 mg/ml in RA1, aliquoted and stored at -80°C until extraction. Total RNA was extracted in duplicate from lysate aliquots using the Nucleospin 96 RNA kit (Macherey Nagel). Contaminating DNA was removed from RNA samples by DNA elution and DNase treatment. Total DNA was recovered from tissue lysates using the Nucleospin 96 tissue kit (Macherey Nagel), according to manufacturer's instructions. PBMCs from non-infected macaques and PBMCs from infected macaques were used as negative and positive controls, respectively. Quantification was performed using an external standard, diluted on a log_10_ scale, to obtain a standard curve as described [32].

### 2-LTR circle analysis using real-time PCR

The 2-LTR junction (≈274 bp) was amplified in duplicate from 250ng of total DNA in a 25 µl reaction mixture comprising 1× Absolute™ QPCR SYB® Green Mixes (ABgene, Surrey, UK), and 400 nM of each primer, REVN1 5′-CTCCTGTGCCTCATCTGATACA-3′ (22 bp) [33] and FMAN 5′-TGTGTGTTCCCATCTCTCCT-3′ (20 bp), which recognizes sequences in the U5 (nt 158–179) and U3 (nt 10162-10181) regions of SIVMM239, respectively. PCR cycles included a denaturation step of 10 min at 95°C followed by 50 cycles of 95°C for 10 s, 61°C for 10 s, and 72°C for 20 s. The copy number of 2-LTR circles was determined in reference to a standard curve generated by PCR amplification of a serial dilution of the plasmid PCR4TOPO2-LTR (containing the SIV_mac251_ 2-LTR junction). DNA sequence analysis of random samples confirmed that the PCR products spanned the 2-LTR junction (Figure S1). In our system (iCycler v3.1, Biorad), we performed linear regression over the 7-log_10_-unit range of the 2-LTR circle standard, which detected up to 20 copies of 2-LTR within 250 ng of cell DNA (**Figure S1**) [11].

### Viral RNA quantification in tissue

RNA extracted from tissue was analyzed in duplicate by RT-qPCR using the Super Script III platinum one-step quantitative RT-PCR system (Invitrogen, Cergy-Pontoise, France) with SIV gag primers and probe as previously described [34]. Reactions and data acquisition were carried out with the I-Cycler real-time PCR system (Biorad). To normalize the RNA input, GAPDH RNA was simultaneously quantified using a previously published primer set and probe [35], [36]. To determine the amplification efficiency, we included negative controls and serial 10-fold dilutions of SIV and GAPDH RNA for each experiment. As the efficiency of each GAPDH and SIV reaction was similar, we conducted a 2^−ΔCt^ analysis. Results were expressed as SIV RNA copies/GAPDH RNA copies.

### Immunohistochemistry (IHC) on paraffin-embedded tissues

IHC was performed on deparaffinized LN, rectum and lung sections, after high-temperature antigen retrieval in the presence of 10 mM sodium citrate buffer, pH = 6, or 1 mM EDTA pH = 9, with primary mAbs recognizing CD3, CD4 and CD68. Clones, isotypes, sources, and conditions were 1F6, IgG1, (Novocastra, Newcastle, UK), pH 9 for CD4; and KP1, IgG1, (Dako, Glostrup, Denmark), pH 9 for CD68, respectively. Ab binding was visualized with the StreptABComplex/HRP duet kit and DAB (3,3 Diaminobenzidine) (Dako). After washing, slides were counterstained with Mayer's Hemalun for 45 sec, and were then dehydrated and mounted. Whole brightfield microscope images (magnification 10× or 4×, area 5 to 100 mm2) per sample were captured with a DS-Ri1 CCD camera mounted on an upright microscope 90i (Nikon Instrument Europe BV, Amstelveen, The Netherlands).

### Quantification of the areas that underwent immunohistochemical staining

To quantify the percentage of the areas stained for CD4 and CD68, images were processed and quantified with ImageJ, a public domain Java image processing program (U.S. NIH). Briefly, brown-colored images specific for H-DAB stain (red = 0.7110272, green = 0.42318153, blue = 0.5615672) were extracted by color deconvolution [37], and measured for specific staining using threshold ImageJ internal commands. The number of pixels corresponding to CD4 or CD68 staining and total tissue was obtained, generating the percentage of the area stained. This percentage, determined using two fields per animal, was then used for the analysis.

### Statistical analysis

Statistical analysis was carried out using nonparametric Wilcoxon and Mann-Whitney rank tests, which are adapted to small sample sizes, using StatView software (SAS Institute Inc., Cary, N.C). Viral production over the course of a selected period of time was measured by computing the area under the curve (AUC) using the trapezoid calculation.

## Supporting Information

Figure S1 Scheme of 2 LTR quantification using Q-RT-PCR and sequences of 2-LTR junction in PCR products cloned into the plasmid used to provide references curves.
(2.53 MB TIF)
