# Supplementary figures and images for: Antiretroviral Treatment Start-Time during Primary SIVmac Infection in Macaques Exerts a Different Impact on Early Viral Replication and Dissemination
Source: PLoS One. 2010 May 11;5(5):e10570. doi: 10.1371/journal.pone.0010570 (PMC2868019; doi:10.1371/journal.pone.0010570)

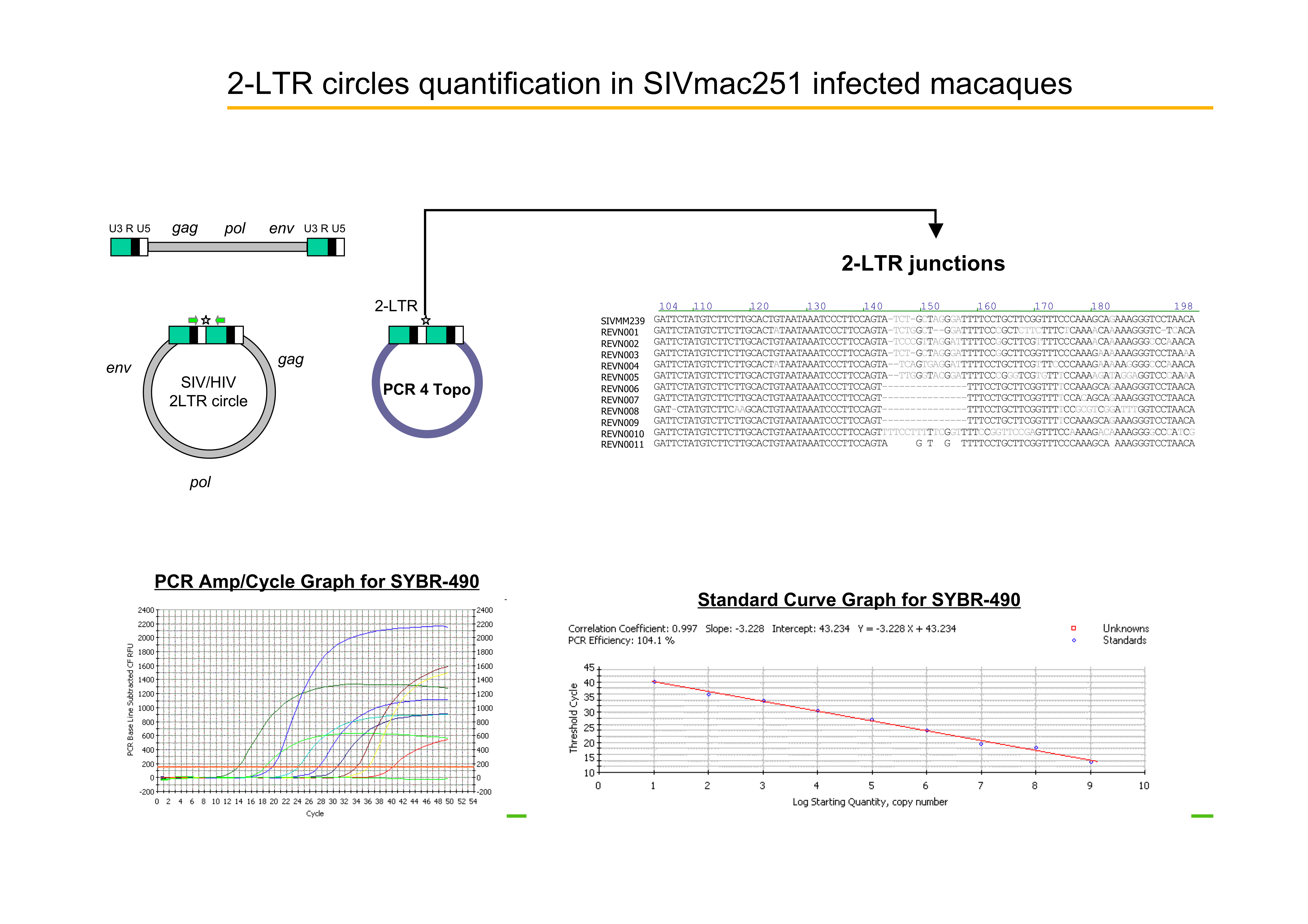

Supplement: Figure S1 — Scheme of 2 LTR quantification using Q-RT-PCR and sequences of 2-LTR junction in PCR products cloned into the plasmid used to provide references curves. (2.53 MB TIF) [file pone.0010570.s001.tif]
